# Management of Acute Organic Change of Character cases by Liaison Psychiatry Unit

**DOI:** 10.1192/j.eurpsy.2024.1017

**Published:** 2024-08-27

**Authors:** E. Cesari, H. Álvarez, H. Andreu, L. Bueno, O. De Juan, J. I. Mena, I. Ochandiano, L. Olivier, S. Salmerón, L. Pintor

**Affiliations:** Department of Psychiatry and Psychology, Institute of Neuroscience, Hospital Clinic de Barcelona; ^2^Department of Psychiatry and Psychology, Head of Liaison Psychiatry Unit, Hospital Clinic de Barcelona, Barcelona, Spain

## Abstract

**Introduction:**

The Acute Organic Change of Character (AOCC) is an organic mental disorder subtype in which perception, thought, mood and personality impairment predominate. It consists in a change in the individual’s general behaviour or attitude, which is shown to be closely associated with or caused by an underlying organic process, and which is rapidly resolved when the organic noxious agent is eliminated (Pintor et al. Journal of Psychiatry and Psychiatric Disorders 4 (2020): 354-358).

**Objectives:**

To describe the importance of taking AOCC diagnosis into consideration and the role of liaison psychiatrists in AOCC management by presenting two AOCC cases admitted to the Hospital Clinic of Barcelona.

**Methods:**

We retrospectively reviewed two AOCC cases in patients followed by our hospital’s liaison psychiatry unit during the summer of 2023. We also searched for previous case reports of AOCC using a PubMed query.

**Results:**

Case 1: A 50-year-old male who suffered a polytrauma with diffuse axonal injury (DAI). His relatives and the referring medical team observed a change in his behaviour consisting in irritability, suspicion, hostility and impatience. No cognitive impairment nor fluctuation in the described symptoms were observed. At the time of discharge character changes were still present due to DAI slow and unpredictable clinical course. Symptomatic treatment with risperidone 6mg/day and quetiapine 100mg/day was administered achieving a satisfactory clinical response.

Case 2: A 47-year-old woman with type 2 diabetes who suffered an infectious cellulitis that spread causing sepsis. The patient began to appear disruptive with verbose and tangential speech during her admission. No cognitive impairment nor fluctuation in the described symptoms were observed. Symptomatic treatment with risperidone 10mg/day and olanzapine 5mg/day was administered achieving a satisfactory clinical response. At the time of discharge character changes described before were almost resolved.

**Conclusions:**

The clinical presentation of both cases suggested organic mental disorders in which a change in general behaviour predominates. Liaison psychiatrists play a key role in AOCC management by recognizing the clinical pattern, helping if needed with psychopharmacological treatment and ensuring a good understanding of the disorder both by the referring medical team and the patient’s relatives. To our knowledge, it would be of great importance to achieve a better understanding of this clinical condition which to date we consider to be underdiagnosed.

**Disclosure of Interest:**

None Declared

